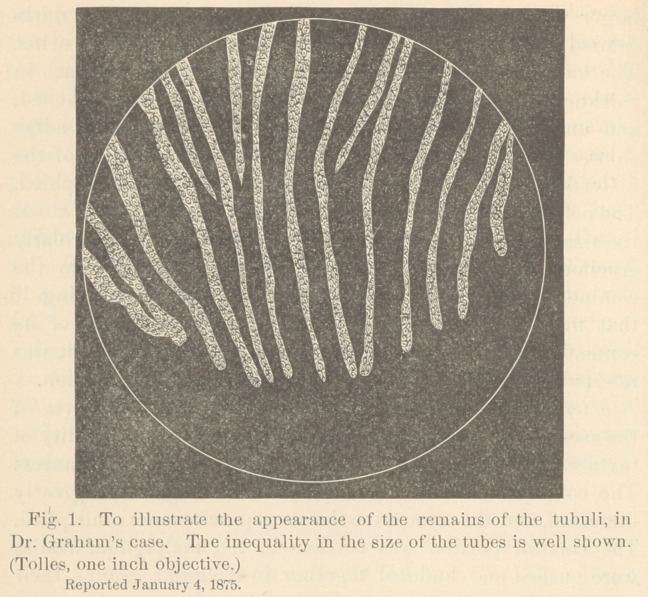# The Pathological Transactions of the Chicago Medical Society

**Published:** 1876-10

**Authors:** 


### THE PATHOLOGICAL TRANSACTIONS OF THE CHICAGO MEDICAL SOCIETY—No. 1.

Edited by Dr. I. N. DANFORTH.

### EXPLANATORY.

At its regular meeting, held on the evening of June 5, 1876, the "Chicago Medical Society" voted to publish its Pathological Transactions, and the undersigned was, with gratifying unanimity, elected editor. In carrying out this plan it is expected that a two-fold object will be accomplished, namely: *First*—to induce the members of the Society to promptly and accurately report their interesting cases, especially those which are accompanied by morbid specimens either of *ante* or *post-mortem* derivation; and, *Second*—to permanently record such cases, so that they shall be accessible to the profession at large for present profit, and future reference.

The editors of the Chicago Medical Journal and Examiner having courteously proffered the use of their columns, the Transactions will, for the present at least, be published as an integral part of the Journal and Examiner. The liberality and enterprise of the publishers of the Journal and Examiner will enable the editor of the Transactions to present illustrations to all articles, which will be rendered more valuable thereby.

Copies of the Transactions alone, paged consecutively for future binding, can be obtained of the publishers, Messrs. W. B. Keen, Cooke & Co., at a cost of twenty-five cents per copy.

The Transactions will appear as often as circumstances require, or as often as a sufficient amount of really valuable material can be secured. For the present it is probable that the numbers will be issued at intervals of about three months. It will be the aim of the editor to make the Transactions a repository of valuable and, so far as possible, original pathological knowledge. The value of researches in the domain of pathology and pathological histology is no longer a matter of doubt. The Chicago Medical Society recognizes this fact; and in commencing the publication of its Pathological Transactions it does but give expression to its desire to do its part in hastening the day when the science of medicine shall be a *science* indeed.

In conclusion the editor earnestly solicits the cheerful and constant support and assistance of the members of the Society, without which his own efforts must necessarily prove both futile and fruitless, but with which much valuable work can be done.

I. N. DANFORTH.

I.

### OCCLUSION OF THE URETER BY A CALCULUS.

By DR. D. W. GRAHAM.

This specimen was accidentally found while eviscerating a subject in the dissecting room some two weeks after it had been received. The subject was a female, aged probably thirty-five years, with an average amount of adipose tissue. It was impossible to get the facts in regard to the history of the patient or the particulars of her illness. But we have the meager information that she was confined, and taken with puerperal fever, of which she died. So far as could be ascertained she had never complained of any symptoms indicating renal disease. Those who made the autopsy had evidently not been led to suspect any disease of those organs, for all the thoracic and pelvic viscera, and all the abdominal except the kidneys, had been thoroughly examined; they remained in situ. There was considerable hypertrophy of the right kidney, but otherwise its gross appearances were normal. The left kidney was so altered in outline and consistence that it was with difficulty recognized. It consisted of a pear-shaped sac, not much larger than the normal kidney, which was filled with a dark-colored fluid, and this fluid had the odor of decomposing urine. The walls of the sac were from one-quarter to one-half inch in thickness in different portions. The capsule was thickened, and united to surrounding structures by inflammatory adhesions. The connective tissue beneath the mucous lining of the distended pelvis of the organ was considerably hypertrophied. The pelvis contained three urinary calculi — two of them about the size of beans, one as large as a chestnut, but irregularly cruciform, the long limb of the cross being inserted into the commencement of the ureter, and so completely closing it that firm pressure upon the kidney failed to empty it of its contents. After laying the sac open and evacuating it, it was referred to Dr. I. N. Danforth for microscopic examination.

*Microscopy.*—Sections were made from various parts of the sac, whose walls varied much in thickness and solidity of texture. I noticed the following microscopic appearances: The connective tissue in all parts of the organ was greatly increased, and the connective tissue corpuscles were multiplied. The cortical portion was condensed, the malpighian bodies were pushed and huddled together in irregular groups, their capillary plexuses being generally condensed, or possibly quite collapsed, by the proliferation of the endothelium of the capsule. The convoluted tubes were sometimes pinched by pressure of the hyperplastic connective tissue, sometimes distended by the accumulation of their thrown off lining cells; hence they were of very unequal caliber. The pyramids were nearly obliterated, and the straight tubes were shortened, distorted, or doubled upon themselves by pressure. In one place I found what I thought might be the remains of a pyramid, and upon making sections I could recognize the straight tubes, in a comparatively efficient condition. I present a camera drawing of a portion of one of the sections, the intertubular connective tissue being omitted. These tubes could be traced upwards until they became convoluted, and in this portion of the organ the glomeruli were but little changed. The coats of the vessels were generally thickened; and this was particularly noticeable in the tunica adventitia. I. N. D.

Fig. 1. To illustrate the appearance of the remains of the tubuli, in Dr. Graham's case. The inequality in the size of the tubes is well shown. (Tolles, one inch objective.)

Reported January 4, 1875.

### II.

### ADENOID (HODGKIN'S) DISEASE.

By Dr. Chas. Warrington Earle.

Mark L. Covell, aged twenty-six, died November 20, 1875. He was under my professional care sixteen days. During that time his disease was characterized by enlargement of the cervical, axillary and inguinal glands, extreme dyspnœa, effusions into the serous cavities, and rapidly advancing anæmia.

At my first visit I learned that some ten days before he had been seized with a terrible paroxysm of dyspnœa, for the relief of which the nearest physician was summoned. The disease was thought to be of a serious pulmonary character, and the patient was advised to visit Colorado. At this time I was requested to see the case. I found him sitting up, free from pain, pulse at one hundred, temperature normal. His respiration, however, was greatly accelerated, and his face and neck presented a most remarkable appearance — the former being puffy and cyanotic, and the anterior and lateral portions of the latter so swollen that at first glance one would say that there was a tremendous enlargement of the thyroid gland. Upon examination, however, I found all the superficial veins of the head and neck enormously distended with blood, as were also the capillaries, giving the cyanotic hue mentioned above, while on the anterior part of the chest a large number of small ecchymosed spots could be seen, which were the result of small ruptured blood vessels. Wherever a lymphatic gland could be detected in the neck or axilla, it was found to be enlarged and indurated, but *not* tender or inflamed. There was dullness post sternum, respiratory sounds exaggerated in upper part of right lung, and evidence of considerable effusion in right pleura. The appetite was poor, bowels regular; no signs, from careful examination, of any kidney complication.

For three days I administered diuretics and cholagogue cathartics, hoping to diminish the amount of fluid in the chest and relieve the dyspnea. On the fourth day, November 8th, I performed aspiration, and drew away forty-three ounces of a yellow-colored fluid. The patient was now greatly relieved; the lividity of the integuments lessened, the swollen condition largely diminished, the breathing quite natural. The anæmia continued to increase, however; the glands were large and hard, and the effusion into the right cavity rapidly recommenced. Tonics and alteratives were of no avail. November 16th, drew away twenty-five ounces of a sanguineous-colored fluid from same cavity. November 18th, pulse 105, temperature normal, respiration 25. November 19th, pulse 110, temperature normal; evening, pulse very weak and rapid, greatly prostrated, intellect clear. Death next morning.

Autopsy, November 21st; present, Drs. Graham and Van Buren. The body was not markedly emaciated; the superficial enlarged glands could be easily distinguished; the ecchymosed spots upon the anterior part of the chest were plainly visible. The head was not opened. The left lung was healthy; the pleura on this side contained two ounces of slightly yellow serum. The right lung was hepatized to a considerable extent; slight adhesions existed, and in the pleural cavity of this side was found forty-eight ounces of serum and blood. The mediastinal space was filled with an accumulation of enlarged glands, forming one large mass, reaching from the middle of and involving the trachea to the ensiform cartilage, impinging also on both lungs, and on the left pressing against the pericardium and contents. On the left side of this tumor, or more correctly mass of tumors, at the point where it came in contact with the heart and membranes, was a smooth, glistening surface, six inches in length by three in breadth, caused undoubtedly by the constant friction of this organ. Within the pericardial sac was found eight ounces of sero-sanguineous fluid. The liver was somewhat soft, the spleen normal. The mass of glands was referred to Dr. O. C. Oliver for microscopic examination.

*Microscopy.*—The tumor, as received from Dr. Earle, presented the appearance of having been very imperfectly preserved, it having, as I was told, been immersed in "Chloral Hydrate solution;" but however well this chemical preserves animal tissues under proper conditions, it had in this instance partially failed, owing in all likelihood to its volume having been relatively too small. Cubical pieces ¼ inch to ½ inch in size were removed from different portions of the tumor, and immersed in a solution of chromic acid of two per cent. strength. In this they were permitted to remain for about two weeks, when sections were made which were immersed in alcohol diluted with its bulk of water. The specimens were next stained with the ammoniacal solution of carmine, after which the silver staining method was employed; and after a great number of trials and some modifications, a passable result was obtained.

A careful study of the specimens seems to me to warrant the following observations: The change in the mass of lymphatic glands originally composing the tumor did not seem to be one of heterogeneous growth, but rather of hypertrophy. This conclusion is arrived at after a careful study of the microscopic appearances, for these reasons, namely: In no case were the atypical cells characteristic of malignant growths observed. The normal structure did not seem to be greatly altered in its appearance beyond what will be noted hereafter. The connective tissue seemed to be relatively greatly increased, but there were not visible the connective tissue corpuscles, which one would expect to find in abundance, but its substance was infiltrated with lymphoid cells. The lymph spaces presented the appearance in some portions of their course, of having become "clogged" up with the debris of lymphoid cells, together with the cells themselves, leading to a gradual obliteration of their caliber. To sum up, then, it seems that the tumor consisted of hypertrophied glandular tissue, with, perhaps, "sub-acute inflammation" products. The infiltrated connective tissue seems to warrant this hypothesis.

This hasty and somewhat incomplete report is rendered necessary by the condition of the specimen. O. C. O.

Reported February 7, 1876.

III.

### CASE OF ASPERGILLUS NIGRICANS.

By Dr. F. C. HOTZ.

The following history was obtained: Mrs. P., aged 26, of Winona, Minn., while pregnant became hard of hearing, about three years ago; never had any earache or sore throat. Soon after her confinement she was troubled with an itching and burning in her left ear, which did not subside until a little watery secretion was discharged from the external meatus. This attack was repeated almost every third month, but always subsided spontaneously. A change, however, was observed in the character of the discharge; it was watery at first, but afterwards appeared to be whitish, and to contain a granular cheesy matter, and during the past two months a black substance has several times been discharged from that ear. The whole meatus appeared black, as if coated with soot. By injections of tepid water a considerable quantity of a substance was removed which consisted partly of black, membranous shreds, partly of a yellowish-white cheesy matter. Under the microscope the black substance exhibited the characteristic formations of the *aspergillus nigricans*, in full bloom; the cheesy matter proved to be composed of cell-detritus and mycelium fibers, without the sporangia and spores. The thoroughly cleansed wall of the auditory meatus was found to be deprived of the epidermis, and was red and somewhat swollen; the external surface of the membrana tympani was dull and opaque. The watch was not heard even when pressed on the left ear, and the tuning fork placed on the forehead was heard in the right ear only.

April 26, 1876.—Examination made, with the following results: Right ear, watch heard only when pressed against the ear; but after catheterization it was heard at the distance of one-half inch. After two weeks of treatment the right ear could hear the watch at two inches, and the left ear at one-half inch. The left meatus was pencilled over once with a solution of nitrate of silver, thirty grains to the ounce, and olive oil containing ten per cent. of carbolic acid was dropped in three times a day for one week. The external otitis subsided within one week; the fungus did not appear again, nor was there any discovered at the final examination on May 14th.

Reported July 17, 1876.

### IV.

### CASE OF FIBROID TUMOR OF THE UTERUS.

BY DR. MARY H. THOMPSON.

Miss C. A., aged thirty-four years, seamstress, born in Scotland, entered the hospital for women and children May 31st. 1876, because of uterine hæmorrhage. She was rather above medium height, neither spare or corpulent in figure, had light complexion, light brown hair, blue eyes, and sharp features. Her face, lips and tongue were much blanched by hæmorrhage. The left arm was paralyzed in the motor nerves so as to hinder action, in the following manner: the hand could not be flexed upon the forearm, nor the forearm upon the arm; but the arm could be flexed upon the chest, the last movement being all that could be made with the arm. I think the nerves of sensation were not paralyzed in any way. The left leg was so œdematous as to seem a fully developed phlegmasia alba dolens.

Apparently there was an intramural fibroid tumor occupying the whole posterior wall of the uterus, which seemed as large as the fœtal head at term. The os was open enough to admit the ends of two fingers. The menstrual flow began at about thirteen years of age, and was quite regular until four or five years since, when it became more profuse, without the period being lengthened, until the last, after which it never ceased during the remainder of her life. The sensation in the hand was that of cold and numbness. The leg and foot were at times painful; at others they had a pricking sensation.

The hæmorrhage began about five weeks before she entered the hospital, as a profuse flooding, and came on just before that menstrual period was due. The paralysis of the arm occurred about two weeks after the hæmorrhage began, and the œdema of the leg about eight days after the paralysis. The uterine discharge was at times bright red blood, and at others a dark watery secretion, containing small brownish decomposing clots, and had an exceedingly offensive odor.

The treatment was simply that designed to prevent septicæmia, by carbolized vaginal washes, and remedies to support the strength, such as iron, quinine, and good food. Ergot was given in small doses, but was found to be useless. The limb was treated like the puerperal phlegmasiæ.

There was but little change from May 31st to June 7th, when I was called away, and the case passed under the professional care of Prof. John Bartlett, who furnished the following supplementary account:

"After the case of fibroid was entrusted to me, the main symptom was hæmorrhage. The paralysis continued the same to the end; the leg from the time you left, improved and became daily less painful and swollen. Fœtor of the uterine discharge was a constant symptom, and the very great pain induced in the palsied leg by any manipulation, interfered with the disinfecting of the vagina. The patient had but little appetite, and the amount of food taken was less than it otherwise would have been, because of a difficulty of swallowing, which seemed to depend upon a paralysis of certain muscles concerned in deglutition. From day to day she grew paler and thinner, and finally death occurred from asthenia, June 13th. The treatment adopted by me was warmth and gentle friction with liniment, to the leg, and such efforts to nourish and sustain as the depressed condition suggested. Neither opium, ergot, the mineral acids or lead were able, permanently, to stop or materially lesson the flow from the uterus. The presence of the evidences of septicæmia, and the putrid nature of the discharge from the womb, deterred me from the use of the tampon, and similar considerations contra-indicated the use of local astringents. I regard the paralysis as due to embolism."

The *post-mortem* appearances were as follows: A partial examination of the body was made eight hours after death. The thoracic and abdominal organs were normal. Those of the pelvis were natural, except the uterus, which was nearly symmetrical, and the body of which was as large as the fœtal head at term — the whole body being developed into a true fibro-myoma, including the fundus and both walls down to the cervix, one cornu being a little lower than the other. Its external surface measured as follows: The vertical diameter was four and one-half inches; the transverse diameter, from one cornu to the other, four inches. The anterior wall, on a level with the superior extremity of the internal vertical diameter, from the serous to the mucous surface, measured an inch and a quarter; the posterior wall two inches; and the fundus, from the serous to the mucous surface, one inch and three-quarters. The depth of the cavity, including the cervix, (which latter could not have measured more than one inch,) was two and one-half inches. The mucous surface was studded with the open mouths of blood-vessels, which looked like so many severed arteries, the diameter of the largest of these mouths measuring one-eighth of an inch. These were found in greater abundance in the fundus and internal os uteri.

Two months and a half after the death of the patient, the brain was carefully examined by Drs. H. M. Lyman and D. W. Graham. It was well preserved, had undergone no softening, but was hardened a little by the action of the preserving fluid — alcohol and water. No structural lesions or evidences of diseased action were found. The efficient cause of the paralysis, therefore, must have been outside the cranium, and consequently must now remain a matter of opinion rather than of certainty.

Note by the Editor.—A microscopic examination of thin sections of the uterine tumor shows that it belongs to the variety known as "small-celled, spindle-celled sarcoma." Its histology is rather rudely illustrated in "Rindfleisch's Pathological Histology," page 138, Fig. 48. "The characteristic textural element," says Rindfleisch (loc. cit.), "is a short and narrow spindle-cell, with an elongated, roundish nucleus, with or without nucleoli." These little fusiform cells are woven together so as to form a dense, unyielding mass. The pointed ends are "dove-tailed," so that there are practically no interfibrillar spaces. There does not seem to be any intercellular substance, except that occasionally one is met with in which a finely granular deposit in very small quantity can be seen between the larger portions of the cells.

In structure the small spindle-celled sarcomas present a very striking analogy to the inflammatory new formations; and the resemblance is strengthened by the peculiar degenerations to which these tumors are liable. Again, they are far less likely to undergo cancerous metamorphosis than the other forms of sarcoma. These several facts have led some pathologists to believe that the small spindle-celled sarcomas are, sometimes at least, of inflammatory origin; that a long-continued chronic inflammation may result in either a local or diffused hyperplasia of tissue elements.

In Dr. Thompson's case the question may fairly be asked whether the general enlargement of the uterus was not purely a hyperplasia involving the muscular tissue of all parts of the organ.

Reported June 19, 1876.

### V.

### A CASE OF HYDATIDIFORM DEGENERATION OF THE OVUM IN A TWIN PREGNANCY.

By Dr. Norman Bridge, for Dr. R. M. Lackey, of Maywood, Ill.

The patient in this case was a German about thirty-five years of age. She had an angular curvature of the spine from disease of the vertebræ in childhood, but had nevertheless always enjoyed fair health. She had been pregnant once before the present experience, but had aborted at the eighth week. She had been married to her present—the second—husband but nine months. Dr. Lackey was called to her hurriedly, to find her apparently in the ninth month of utero-gestation and bleeding freely. The patient stated that for three months previous to his being called she had frequent profuse hæmorrhage, for which she was treated by a homœopathic doctor. She supposed herself to be only six months advanced in pregnancy. She had been flowing constantly for three days, and had feeble labor pains. She "was much exhausted from loss of blood and nervous irritation."

On digital examination the os uteri was found only slightly dilated, barely enough to admit the index finger. The finger encountered a "soft, lumpy mass that occupied the lower portion of the cavity of the uterus;" through this "no part of a fœtus could be detected." Dr. Lackey wisely judged that the only means of saving the patient's life was to evacuate the uterus of its contents and secure contractions. Accordingly he administered ergot freely, and proceeded to dilate the os with his fingers. As soon as this was accomplished he grasped a handful of the soft mass and brought it away. It was composed of cysts varying in size from that of a pea to that of a pigeon's egg, and filled with a fluid resembling the amniotic liquor. "Each had a pedicle, and was attached to a fibrous cord or stem so as to resemble a cluster of grapes." This *stem* was the only part that was attached to the uterine wall. The specimen, to all appearance, was one of hydatidiform degeneration of the ovum.

Dr. L. continued to bring the mass away in handfuls, until with the last shred it measured over three pints. He discovered in the course of this operation that the uterus also contained what appeared to be a normal bag of water containing a fœtus. He promptly ruptured the bag, when "fully the ordinary amount of water escaped." He at once found the feet, brought them down, and delivered the fœtus. It was apparently six months old, and had evidently been dead several days. The placenta was removed manually; it was normal in appearance. Not until it was removed did the uterus make the slightest effort at contraction; it then contracted sufficiently to stop further hæmorrhage. "The patient was prostrated and exsanguinated after the operation, but gained rapidly and made a fair recovery." Dr. Lackey declares that there was not the slightest connection between the cysts and the placenta, membranes, or any of the normal contents of the womb." The attachment of the cystic mass to the uterine wall was a sufficient distance from the border of the placenta to make the doctor positive in his assertion that the two were in no way connected. Furthermore, he examined the placenta carefully after its delivery, to discover any possible point of connection with the degenerate ovum, and could find none.

This specimen does not differ from any specimen of a cystic degeneration of the ovum. There is nothing peculiar in the crude appearances of the cysts; but the case is interesting and peculiar, if not unique, in that there was evidently an ordinary twin impregnation, one ovum of which soon underwent the well-known cystic degeneration, the other going on in a normal course until the pressure of a large foreign body growing by its side, and the loss of blood resulting from the abnormal condition, led to its death.

The history of this case is made up from notes which Dr. Lackey has kindly furnished.

NOTE.—The specimen presented to the Society by Dr. Lackey consisted of a mass of cysts large enough to about half fill a morphine bottle. They varied greatly in size, the smallest being no larger than a homœopathic pellet, the largest about the size of a common marble. They were all balloon shaped, the small end of the balloon terminating in a long delicate pedicle, by which they were tethered in groups of ten to twenty. After being immersed in glycerine, alcohol and water a few days, the cysts became beautifully transparent, so that their minute structure could be easily studied. The cyst walls were composed of simple membrane, lined with a layer of rather small pavement epithelium-cells. The fluid contents consisted of a clear serum, in which a few granular corpuscles floated, closely resembling the "gorged granules" found in ovarian fluids, except that they were smaller. A careful search was made for the hooklets of echinococci, but with only negative results. True hydatids within the uterus appear to be exceedingly rare, Rokitansky never having seen but a single case in which true acephalocysts were present, and that case still standing alone (?) upon the records. Dr. Lackey's case was one of true hydatidiform degeneration of the villi of the chorion, a change which is said not to occur after the third month of gestation. The appearance of a group of these cysts, and their anatomical relations to the chorion, is admirably shown by an illustration in "Hewitt's Diseases of Women," page 86, Fig. 3. I. N. D.

Reported August 21, 1876.

### VI.

### SUDDEN DEATH FROM OCCLUSION OF THE LARYNX BY A FRAGMENT OF COAL.

BY DR. I. N. DANFORTH.

In the month of June, 1871, I was urgently summoned to see a boy who was said to be "choking to death." Upon arriving at the place indicated, which was but a few blocks from my residence, the boy was dead. From his parents I obtained the following history of the case: The boy was eight years old; he had always been strong, healthy and active; he had no cough or tendency to any difficulty of breathing; indeed, he had been foremost in games and sports among the children of his neighborhood. Not more than half an hour before his death he was sent on an errand to the neighboring grocery, and it was noticed that he carried a "blow gun," which happened to be the toy most in favor at that particular time. A few moments later he rushed into the house, his face distorted by agony and fright; he managed to utter in a hoarse whisper, "Mother, I'm choking," and immediately fell upon the floor. Two or three desperate but ineffectual efforts at inspiration followed, and he was dead. On the following morning I made an examination of the thorax and cervical region, assisted by Drs. A. H. Foster and Norman Bridge. The lungs were perfectly healthy, so far as their own special structure was concerned, but they were congested to the last degree. The right side of the heart was distended by a soft clot of black blood; the left side was empty and almost spasmodically contracted. No hæmorrhage was found in any part of the chest, although it is said to be common in such cases, especially in the pleural and pericardial cavities. The trachea was intensely congested, and contained a large quantity of mucus, but no blood. In all other respects it was perfectly healthy. Upon removing the larynx and looking into it from above, a glistening black body was seen. When the larynx was opened, a fragment of hard coal was found firmly impacted in the laryngeal cavity, below the inferior or true vocal cords. The muscles of the larynx were in a state of intense contraction; in fact, so pronounced was this, that the foreign body could not be moved by means of a pair of dressing forceps, until the larynx was laid open. The fragment was composed of ordinary anthracite coal, of an irregularly cubical form, and presenting several sharp, protruding angles. It measured three-eighths of an inch in its smallest, by almost half an inch in its largest diameter, and weighed six grains. It seems almost incredible that a body so large and irregular could elude the never-sleeping vigilance of the epiglottis, and find its way through the rima-glottidis of a boy so young. A question of great practical interest presented itself in connection with the case, namely: How came the fragment of coal in the child's larynx. As I have already stated, when he went upon his last errand he carried a "blow gun"—apparently an innocent toy, but in this instance an instrument of death. The "blow gun" is a light metallic (usually tin) tube, fifteen or twenty inches long, and about half an inch in diameter. It is a kind of air gun, and is used in this way: A pea or bean is placed in the tube two or three inches from the end, the lungs are then inflated by a forced inspiration, the lips applied closely around the *opposite* end of the tube, and the inspired air is forcibly expelled, or blown through it; hence the name "blow gun." Of course the pea, bean, or other "projectile,"

is shot from the distal end of the tube with considerable force by the current of air, and may be so directed as to hit a target which is only a few feet distant. The danger of the "blow gun" consists in this: that a forced inspiration may possibly "draw" the projectile (whatever it may be) into the air passages, if the proximal end of the gun be held opposite the mouth when the act of inspiration is performed. Any one who has watched a child when he forcibly inflates his lungs, will readily understand this: the forced inspiration of a child is only comparable to the same act in *another* child. It was probably precisely in this manner that the accident I have been describing occurred. The child reached the grocery safely, did his errand, and started for home. On the way he passed a coal office, in front of which several samples stood in show-boxes; from one of these he took a little fragment and placed it in his blow gun, then holding the open end of the gun on a level with his open mouth, he forcibly inflated his lungs, and in so doing drew a rapid current of air through the gun. This column of air swept the piece of coal through the gun tube and into the child's larynx.

Reported September 21, 1876.

## Figures and Tables

**Fig. 1. f1:**